# Etiologic workup in cases of cryptogenic stroke: protocol for a systematic review and comparison of international clinical practice guidelines

**DOI:** 10.1186/s13643-019-1247-6

**Published:** 2019-12-17

**Authors:** Emma P. Bray, Naoimh E. McMahon, Munirah Bangee, A. Hakam Al-Khalidi, Valerio Benedetto, Umesh Chauhan, Andrew J. Clegg, Rachel F. Georgiou, Josephine Gibson, Deirdre A. Lane, Gregory Y. H. Lip, Elizabeth Lightbody, Alakendu Sekhar, Kausik Chatterjee, Caroline L. Watkins

**Affiliations:** 1 Faculty of Health and Wellbeing, University of Central Lancashire, Preston, PR1 2HE UK; 2 Medtronic Limited, Building 9, Croxley Park, Hatters Lane, Watford, WD18 8WW UK; 3 Faculty of Clinical and Biomedical Sciences, University of Central Lancashire, Preston, PR1 2HE UK; 4 Liverpool Centre for Cardiovascular Science, University of Liverpool & Liverpool Heart and Chest Hospital, Liverpool, UK; 5 Aalborg Thrombosis Research Unit, Department of Clinical Medicine, Aalborg University, Aalborg, Denmark; 6 The Walton Centre NHS Foundation Trust, Liverpool, L9 7LJ UK; 7 Countess of Chester Hospital, Liverpool Rd, Chester, CH2 1UL UK

**Keywords:** Cryptogenic stroke, Stroke, Clinical practice guidelines, Diagnosis, Assessment

## Abstract

**Background:**

Stroke is a leading cause of death and disability worldwide. Identifying the aetiology of ischaemic stroke is essential in order to initiate appropriate and timely secondary prevention measures to reduce the risk of recurrence. For the majority of ischaemic strokes, the aetiology can be readily identified, but in at least 30% of cases, the exact aetiology cannot be determined using existing investigative protocols. Such strokes are classed as ‘cryptogenic’ or as a stroke of unknown origin. However, there exists substantial variation in clinical practice when investigating cases of seemingly cryptogenic stroke, often reflecting local service availability and the preferences of treating clinicians. This variation in practice is compounded by the lack of international consensus as to the optimum level and timing of investigations required following a stroke. To address this gap, we aim to systematically review and compare recommendations in evidence-based clinical practice guidelines (CPGs) that relate to the assessment and investigation of the aetiology of ischaemic stroke, and any subsequent diagnosis of cryptogenic stroke.

**Method:**

We will search for CPGs using electronic databases (MEDLINE, Health Management Information Consortium (HMIC), EMBASE, and CINAHL), relevant websites and search engines (e.g. guideline specific websites, governmental, charitable, and professional practice organisations) and hand-searching of bibliographies and reference lists. Two reviewers will independently screen titles, abstracts and CPGs using a pre-defined relevance criteria form. From each included CPG, we will extract definitions and terms for cryptogenic stroke; recommendations related to assessment and investigation of the aetiology of stroke, including the grade of recommendations and underpinning evidence. The quality of the included CPGs will be assessed using the AGREE II (Appraisal of Guidelines for Research and Evaluation) tool. Recommendations across the CPGs will be summarised descriptively highlighting areas of convergence and divergence between CPGs.

**Discussion:**

To our knowledge, this will be the first review to systematically compare recommendations of international CPGs on investigating the aetiology of ischaemic stroke. The findings will allow for a better understanding of international perspectives on the optimum level of investigations required following a stroke and thus contribute to achieving greater international consensus on best practice in this important and complex area.

**Systematic review registration:**

PROSPERO CRD42019127822.

## Background

Globally, stroke is the second most common cause of death [1] and the third most common cause of disability [2]. In the UK alone, the direct and indirect costs of stroke are around £26bn per year [3]. Those who survive an initial stroke are at high risk of further stroke [4], and a quarter of all stroke survivors will have a further stroke within 5 years [5]. These subsequent strokes are associated with worse outcomes and an even higher mortality than the initial stroke [6]. It is therefore essential that the aetiology of a stroke can be identified, not only to guide treatment for specific conditions, but also to initiate appropriate and timely secondary prevention to reduce the risk of recurrence [7].

Although most ischaemic strokes are caused by atherosclerosis or arteriosclerosis of the blood vessels within the brain, or embolism originating from the heart or the major extracranial blood vessels, in at least 30% of cases, the exact aetiology of an ischaemic stroke is not identifiable by existing investigative protocols [8]; this is classed as a cryptogenic stroke or stroke of unknown origin. Classifying an ischaemic stroke as ‘cryptogenic’ is therefore based on the exclusion of other well-established causes of stroke [9], and with more than 200 possible causes of a stroke, arriving at this classification can pose a formidable challenge to clinicians [10]. Standard diagnostic workup of patients tends to include brain imaging, vascular imaging and cardiac evaluations [8]. If these investigations fail to identify the aetiology of the stroke, further investigations tend to depend on local service availability and on the preference of the treating clinician. Therefore, a ‘diagnosis’ of cryptogenic stroke is often allocated loosely in the absence of a thorough workup, rather than the cause truly being unknown. This has important implications especially in light of the lack of available options for secondary prevention for strokes of unknown source. For example, two recent randomised trials of non-vitamin K antagonist oral anticoagulants did not show a reduction in recurrent stroke compared to aspirin [11, 12], and one of these showed possible harm with an excess of bleeding [11]. This is in contrast to the well-established benefits for stroke prevention with oral anticoagulation in patients where a cardiogenic cause such as atrial fibrillation is found [13]. Patients whose stroke is labelled as cryptogenic are unable to take steps to reduce their risk of a subsequent stroke.

Diagnostic workup in cases of seemingly cryptogenic stroke is highly complex, and from existing guidelines, it is difficult to discern specific recommendations for diagnostic pathways. Additionally, up to now, only one diagnostic algorithm has been proposed to classify cryptogenic stroke [10]. This algorithm has identified three levels of investigation in an hierarchical fashion : (1) standard level, which may be considered the absolute minimum, (2) advanced level, and (3) highly specialised level where an even more advanced set of investigations may be performed. These recommendations are in line with the American Heart Association–American Stroke Association and American Academy of Neurology guidelines [14–17].

As such, while some guidelines and published diagnostic algorithms have been proposed to facilitate the identification of the aetiology of ischaemic stroke, there does not yet exist international consensus on the optimum level and timing of investigations required following a stroke. Therefore, we aim to review international clinical practice guidelines (CPGs) to compare recommendations and pathways for the diagnostic workup necessary before arriving at the diagnosis of cryptogenic stroke, to understand where there are agreement, disagreement and conflicting recommendations. The review findings will inform prioritisation of future research in this emerging field of inquiry.

### Aim

The aim is to compare and appraise recommendations in evidence-based international clinical practice guidelines (CPGs) that relate to the assessment and investigation of the aetiology of ischaemic stroke and any subsequent diagnosis of cryptogenic stroke.

## Methods

The review has been designed based on input from experts in stroke and systematic review methodology and in accordance with the Preferred Reporting Items for Systematic review and Meta-Analysis Protocols (PRISMA-P) guidance. A PRISMA-P reporting checklist is provided in Additional file 1. The review is registered on PROSPERO, number: CRD42019127822.

### Eligibility criteria

We will include CPGs that

1. Have been endorsed by a national and/or international organisation (e.g. governmental, charitable and professional practice)
2. Include recommendations related to the assessment and investigation of the aetiology of ischaemic stroke and any subsequent diagnosis of cryptogenic stroke
3. Were published from January 2009 onwards (to ensure only the most up-to-date guidelines were included)
4. Are available in English

CPGs focused exclusively on treatment and rehabilitation of stroke will be excluded.

### Information sources and search strategy

Relevant CPGs will be retrieved using a combination of search strategies: (1) searching electronic databases, (2) searching relevant websites, (3) targeted searches by country and (4) hand-searching reference lists of included guidelines. Medical Subject Headings and text words such as ‘clinical practice guidelines’ and ‘stroke’ will be used to search electronic databases MEDLINE, Health Management Information Consortium (HMIC), EMBASE, and CINAHL. A draft search strategy for MEDLINE is provided in Additional file 2. As CPGs are often not included in electronic databases, we will also systematically search for guidelines using popular search engines and relevant websites. These websites will include, but are not limited to, the American Academy of Neurology website (https://www.aan.com/), the Cochrane Library, Guidelines International Network (www.g-i-n.net); National Institute for Health and Care Excellence (https://www.nice.org.uk/), Scottish Intercollegiate Guidelines Network (https://www.sign.ac.uk/) and along with grey literature repositories (e.g. Open Grey (http://www.opengrey.eu/)). We will use keywords in the search utilities of each website to identify potentially relevant guidelines and conduct targeted searches by country. We will screen all relevant results retrieved through these searches. A final list of all relevant CPGs identified through this search strategy will be shared with the expert steering group to identify any additional CPGs that could contribute to the review aims and/or confirm that no relevant CPGs, of which the steering group members are already aware, have been omitted.

### Study selection

One reviewer (NMcM) will collate all retrieved citations into reference management software for screening. Two reviewers (NMcM/MB) will independently screen all retrieved citations using a pre-defined relevance criteria form. For potentially relevant CPGs, the full texts will be obtained and independently assessed by two reviewers (from NMcM/MB/EB/RG/JG), including at least one with extensive experience of working in a clinical role in stroke care. Disagreements at any stage will be resolved through discussion with a third reviewer.

### Data collection process and data items

A bespoke data extraction form will be developed, piloted and modified as necessary. One reviewer will extract all relevant information using this form. A second reviewer will perform a full check of the data extraction for all included guidelines to verify the accuracy of data extraction. Any disagreements will be resolved through discussion with a third reviewer. We will extract the following information from each CPG: year of dissemination; country/region; organisation; development team; funding; definition or terms used to describe cryptogenic stroke; recommendations related to assessment, investigation and diagnosis of stroke aetiology (including the recommended timing and order of investigations); recommendations related to the diagnosis of cryptogenic stroke; research evidence cited; and qualifying terms (such as level of evidence or grade of recommendation) used to summarise the strength of recommendations and their underpinning evidence.

### Quality appraisal

We will appraise the quality of the included CPGs using the Appraisal of Guidelines Research and Evaluation (AGREE) II [18] tool which assesses the scope and purpose, stakeholder involvement, rigour of development, clarity and presentation, applicability and editorial independence. As recommended, each guideline will be independently assessed by four reviewers [19], and average ratings calculated to produce a quality score for each of the six domains. In line with similar reviews, we will assess agreement for each domain item and collectively review items where appraisers scores were > 1.5 standard deviations (SD) from the mean item score [20]. A domain will be considered to be adequately addressed if scoring ≥ 60% [20–23].

### Synthesis

Recommendations related to assessment, investigation and diagnosis of stroke aetiology and diagnosis of cryptogenic stroke will be summarised descriptively. We will evaluate whether guidelines made specific recommendations and, in the cases where this is done, we will describe the grade of the recommendation, the level of evidence supporting the recommendation, and where the level of details allows, the nature and quality of studies supporting/refuting the recommendation. We will organise the identified recommendations into categories using a recently published identification and diagnostic evaluation algorithm [10] and produce summary tables to illustrate where there is consensus or disagreement between recommendations presented in the individual CPGs. Finally, we will compare the recommendations identified in each of the CPGs with the diagnostic evaluation algorithm to assess the extent to which there is agreement between each recommendation [10].

## Discussion

This review forms part of a wider programme of work exploring the optimisation of assessment and investigation of the aetiology of ischaemic stroke and the diagnosis of cryptogenic stroke. To our knowledge, this will be the first systematic review to compare the content of recommendations in relevant international CPGs. The review findings will allow for a better understanding of similarities and differences in international recommendations for identifying the aetiology of a stroke, the optimum level of investigations required following a stroke and the quality of evidence underpinning practice recommendations. Additionally, the review findings will have practical implications for the detection and management of patients with cryptogenic stroke. This review will follow a robust systematic method to identify all relevant publications. It will however only include English language publications which are a limitation.

## Supplementary information

**Additional file 1.** PRISMA-P-checklist

**Additional file 2.** MEDLINE search strategy

**Additional file 3.** Confirmation of funding

## Data Availability

Not applicable

## Abbreviations

AGREE: Appraisal of Guidelines Research and Evaluation
CPG: Clinical practice guideline
ID-CRYPT: Investigating the Detection of CRYPTogenic stroke
LCCG: Liverpool Clinical Commissioning Group
NHS: National Health Service
NIHR CLAHRC NWC: National Institute for Health Research Collaboration for Leadership in Applied Health Research and Care North West Coast
NOACs: Non-vitamin K oral anticoagulants
OAC: Oral anticoagulation
PRISMA-P: Preferred Reporting Items for Systematic Review and Meta-Analysis Protocols
