# Supplementary material for: Etiologic workup in cases of cryptogenic stroke: protocol for a systematic review and comparison of international clinical practice guidelines
Source: Syst Rev. 2019 Dec 17;8:331. doi: 10.1186/s13643-019-1247-6 (PMC6918649; doi:10.1186/s13643-019-1247-6)
Supplement: Supplementary file 2 — Additional file 2. MEDLINE search strategy [file 13643_2019_1247_MOESM2_ESM.docx]

**Additional file 2: MEDLINE search strategy**

| **1** | exp Guideline/ |
| --- | --- |
| **2** | exp Practice Guidelines/ |
| **3** | (guideline or guidelines).ti |
| **4** | 1 or 2 or 3 |
| **5** | exp Stroke/ |
| **6** | stroke.mp |
| **7** | 5 or 6 |
| **8** | 4 and 7 |
