# Supplementary material for: Etiologic workup in cases of cryptogenic stroke: protocol for a systematic review and comparison of international clinical practice guidelines
Source: Syst Rev. 2019 Dec 17;8:331. doi: 10.1186/s13643-019-1247-6 (PMC6918649; doi:10.1186/s13643-019-1247-6)
Supplement: Supplementary file 3 — Additional file 3. Confirmation of funding [file 13643_2019_1247_MOESM3_ESM.pdf]

NHS
Liverpool
Clinical Commissioning Group

NHS Liverpool CCG
The Department
2 Renshaw Street
Liverpool
L1 2SA
Tel: 0151 296 7654
Email: dave.horsfield@liverpoolccg.nhs.uk

University of Central Lancashire
Preston
PR1 2HE

Date: 22.11.2018

Dear Professor Watkins

Liverpool Clinical Commissioning Group (LCCG) RCF Award 2018/19

Thank you for your application for funding from the recent LCCG RCF Call.

On behalf of the LCCG RCF Funding Panel, I have pleasure in offering the University of Central Lancashire, the **"Grant Holder"** a grant of **£24,948** excluding VAT **("the Funding")**, for the research proposal **Investigating the Detection of CRYPTogenic stroke (ID-CRYPT)** (**"the Research"**), a copy of which is attached as Appendix 1 to the letter. The grant shall take effect from **1st February 2019 and shall continue until 31st July 2019**. Unless terminated in accordance with paragraph 6 below.

The terms and conditions of the offer as follows:

1. Payment shall be made as highlighted in Appendix 2.

2. Please ensure the allocated Purchase Order (PO) number is quoted on all invoices, otherwise the invoices will not be paid. A copy of the PO will be sent in due course.

3. Offers of the Funding are conditional on the Grant Holder obtaining any necessary ethical approval and identifying a sponsor (as defined in the UK Policy Framework for Health and Social Care Research and/or the UK Clinical Trials Regulations as appropriate) for the Research.

4. The Funding is to be used solely in respect of the specific study for which the grant is awarded. This can only be changed with the specific prior approval of the LCCG R&D Department before any expenditure incurred on the amended research. We reserve the right to withhold a grant or require repayment if the work undertaken is not the work for which the grant was approved.

5. Any proposed amendments to the use of the Funding (virement of funds) during the course of the Research must be referred to the LCCG R&D Manager for approval prior to such changes being initiated.

6. Any part of the grant that is not required for the purpose approved will be refunded to the LCCG for use in further projects supported by the LCCG.

7. The start of the Research may only be delayed with LCCG R&D Department agreement.

8. A schedule for submitting progress reports and agreeing dates for progress meetings should be agreed with the LCCG R&D Manager and the Grant Holder.

9. A discussion regarding any NIHR bids and potential hosting arrangements which result from the Research should be had with the LCCG R&D Manager well in advance of submission.

10. The LCCG R&D Strategy Group shall be kept informed of the progress made on the Research and the Grant Holder will be asked to provide progress reports during the Research in the format available from the LCCG R&D Manager.

11. The Grant holder should ensure that any publications, presentations or any other printed materials carry acknowledgements to the financial support of LCCG. The Grant Holder will be obliged to furnish the LCCG R&D Strategy Group with copies of any publications that arise from the Research, including presentations at conferences, at least fourteen days in advance of the submission of such proposed publication to a journal editor or other third party. For presentations for conferences or similar, the Grant Holder agrees to include a disclaimer to state that all views are that of the Grant Holder and not the views of the LCCG. The LCCG R&D Department must be contacted if the Grant Holder wants to acknowledge LCCG’s grant in a press release or any other type of media coverage.

12. The Grant Holder agrees to treat any information that it learns about the LCCG in the course of undertaking the Research, secret and confidential. This applies to any information which ought to reasonably be classed as “Confidential Information”, The following should not be treated as Confidential Information: any information which comes into the public domain through no act or default of the Grant Holder, its officers, agents or employees, or any information which is required to be disclosed by law, or has been disclosed by a third party at liberty to disclose such information without breaching the terms of this agreement.

13. The Grant Holder grants to LCCG a non-exclusive, royalty-free license to use intellectual property (the IP) generated as a result of the Funding. Should the Grant Holder wish to use the IP for any other reason than the furtherance of research, it shall notify LCCG of the same stating this intended purpose.

14. LCCG are willing to accept a request for a reasonable extension should the Grant Holder believe they require one. This request needs to be made in an appropriate timeline and form to ensure that research obligations can be met.

15. The LCCG accept no responsibility or liability, financial or otherwise, arising from the Research. Nothing in this Agreement shall operate to exclude or restrict the Grant Holder’s or LCCG’s

liability for: death or personal injury caused by negligence, fraud or fraudulent misrepresentation, or any other liability which may not be excluded or restricted by law.

16. Notwithstanding that the Grant Holder will perform the Research in line with Appendix A and outputs of this agreement, the Grant Holder does not guarantee nor makes a representation to LCCG that specific results, materials or intellectual property will arise from the Research.

17. No variation or amendment to this agreement will be effective unless it is made in writing and signed by a representative of each party.

All communication with respect to this grant shall in the first instance be with the LCCG R&D Manager, Gabrielle Marr, telephone number 0151 296 7726, email gabrielle.marr@liverpoolccg.nhs.uk

If these terms are acceptable, please have both copies of the agreement signed by an authorised signatory of your University and return one copy to me at the email address below.

Yours sincerely

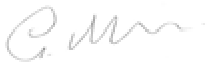

Gabrielle Marr
Research and Development Manager
NHS Liverpool CCG

Enc.

For and on behalf of The Grant Holder:

Name: NIGEL HARRISON

Job Title: EXECUTIVE DEAN

Signature: NHarrison

Date 31.01.19

Acknowledged by the Principal Investigator:

Name: Professor Dame Caroline Watkins

Job Title: Director of Research and Innovation

Signature:

Date: 01/02/19

Appendix 1: Proposal document.

Proposal attached and agreed response to peer review comments.

Appendix 2: Schedule of payments

Invoice to be submitted ASAP	£ 24,948
**Total**	**£ 24,948**
